# Changes in the Gastrointestinal Microbiota Induced by Proton Pump Inhibitors—A Review of Findings from Experimental Trials

**DOI:** 10.3390/microorganisms12061110

**Published:** 2024-05-30

**Authors:** Reidar Fossmark, Maya Olaisen

**Affiliations:** 1 Department of Clinical and Molecular Medicine, Faculty of Medicine, Norwegian University of Science and Technology (NTNU), 7030 Trondheim, Norway; maya.olaisen@stolav.no; 2 Centre for Obesity Research, Clinic of Surgery, St. Olav’s University Hospital, 7030 Trondheim, Norway; 3 Medicus Endoscopy, 7042 Trondheim, Norway; 4 Department of Gastroenterology, St. Olav’s Hospital, Trondheim University Hospital, 7030 Trondheim, Norway

**Keywords:** proton pump inhibitors, acid secretion, microbiome, microbiota

## Abstract

The use of proton pump inhibitors (PPIs) has increased considerably in many Western countries, and there is concern that numerous conditions and diseases associated with PPI use may be adverse events. The main function of gastric acid is to defend the organism against orally ingested microorganisms, and there is also concern that alterations not only in the gastric microbiome but also the downstream intestinal microbiome may increase the risk of disease or alter the course of preexisting disease. The current study is a systematic review of the available evidence from experimental trials investigating the effects of PPIs on the gastrointestinal microbiota by next-generation sequencing. Thirteen studies were identified. The effects of PPIs were seen on alterations in diversity and richness in some of the studies, while a larger proportion of the studies detected alterations at various taxonomic levels. The general finding was that PPI use caused an increase in bacteria normally found in the oral microbiota in both the upper and lower GI tract. The most consistent taxonomic alterations seemed to be increases in oral flora along the axis Streptococcaceae and *Streptococcus* at genus level and various *Streptococcus* spp., as well as Veillonellaceae, *Veillonella* and *Haemophilus*.

## 1. Introduction

The main function of gastric acid is to reduce the number of microorganisms that reach the small intestine. Gastric acid is also essential in the pathogenesis of esophagitis and peptic ulcer disease, and the efficient inhibition of gastric acid secretion by proton pump inhibitors (PPIs) is a cornerstone in the medical treatment of these diseases. However, PPIs have increasingly been used against a range of symptoms and conditions, often with limited evidence of beneficial effect, and high proportions of the population in Western countries now use PPIs [1,2,3]. While the primary purpose of PPIs is to treat acid-related conditions such as acid reflux and peptic ulcers, PPIs can also alter the gut microbiota. This is of great importance, since numerous diseases that may be caused by alterations in the gastrointestinal (GI) microbiome have been associated with PPI use in epidemiological studies.

High-throughput sequencing (also named next-generation sequencing (NGS)) methods have revealed that the majority of GI bacteria are not detected by culture-based methods [4]. NGS methods have since dominated the research on the GI microbiome in health and disease.

Numerous cross-sectional studies have reported that the GI microbiota of PPI users differs from that of non-users in diversity and composition at various taxonomic levels [5,6,7,8]. Although such observational studies suggest how PPI use may alter the GI microbiota, only a few experimental studies of high quality have been published. In the current review, we aimed to describe the changes in GI microbiota reported in experimental studies.

## 2. Materials and Methods

**Figure 1:**
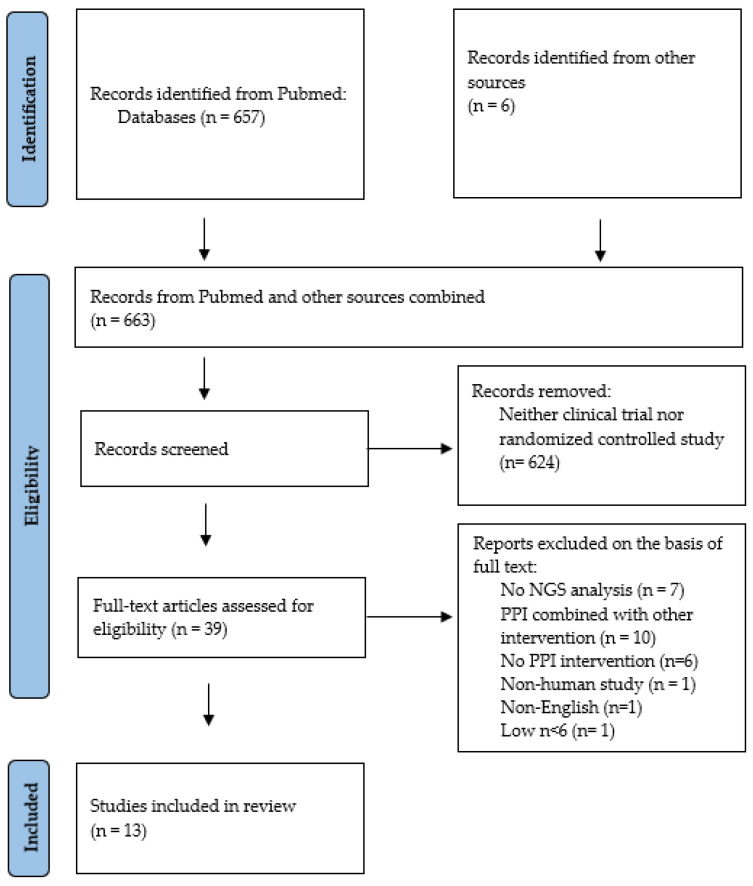
Flowchart illustrating the identification of experimental trials where changes in gastrointestinal microbiome induced by proton pump inhibitors were investigated. The flowchart was adapted from Page et al. [9].

A database search in PubMed until 1 February 2024, using the search terms ((PPI OR (proton pump)) AND (microbiome OR microbiota)), yielded 657 studies where the abstracts were screened by both authors to identify clinical trials in which the microbiota of PPI users was studied. In addition, the reference lists of eligible publications were searched for potentially missed studies. A total of 39 full-text publications were then further assessed. Studies without microbiome analyses, non-NGS methodology, PPI administered in combination with another intervention, low number of study participants in analyses (defined as n < 6), non-English publication and non-human studies were excluded. Discussions were held to address disagreements before reaching a conclusion. Thirteen studies met the pre-specified criteria and were included in the further review (Figure 1). The following variables were recorded: study design, number of participants, PPI dose and duration of use, biological sample type, DNA extraction method, DNA region targeted, sequencing platform, quality filtering and reference database.

## 3. Results

The thirteen studies with an interventional design that investigated PPI-induced gastrointestinal microbiome alterations are presented in Table 1. A total of 316 participants using PPI were included in the reviewed studies.

### 3.1. PPI-Induced Changes in the Microbiota of the Upper GI Tract

Two studies described changes in the microbiota in the upper GI tract. Amir et al. [10] studied patients with gastroesophageal reflux disease (GERD) symptoms before and after treatment with lanzoprazole 30 mg × 2 for 8 weeks. The microbiota in esophageal biopsies and gastric fluid was characterized by differences in β-diversity as well as differences in several taxa at family level. In esophageal biopsies, the Clostridaceae, Lachnospiraceae, Microccocaceae and Actinomycetaceae families were increased, whereas Erysipelotrichaceae was increased in gastric fluid. Among these described changes, Microccocaceae were also upregulated in the fecal microbiota in the study performed by Freedberg et al. [11]. A study by Mishiro et al. [12] examined the microbiota in saliva and in the periodontal pocket. They found reduced α-diversity in the saliva and an increase at genus level in *Veillonella* and *Neisseria* and an increase in *Leptotrichia* in periodontal pocket samples. These genera did not correspond to the families with increased abundances in esophageal biopsies and gastric fluid found by others [10].

### 3.2. PPI-Induced Changes in the Microbiota of the Lower GI Tract

Eleven studies investigated PPI-induced changes in the fecal microbiota, while the microbiota in mucosal biopsies of the rectum or colon has not been investigated. Seto et al. [13] examined nine healthy volunteers at days 0, 7, 28 and 56 after randomization to omeprazole 20 mg × 1 or 20 mg × 2 for 4 weeks and found reduced α-diversity (observed operational taxonomic unit (OTU) count), whereas no significant differences at any taxonomic level were found. As pointed out by the authors, the lack of taxa-specific associations could be caused by heterogeneities at baseline that included differences in age, environment, diet and native microbiomes and that an additional PPI effect was difficult to detect within a small sample size. Samples from the same subjects remained more similar to each other than to those from other participants, indicating that the main microbial communities were not influenced during PPI use.

Bajaj et al. [14] studied 30 participants, 15 patients with cirrhosis and 15 age-matched healthy controls, before and after two weeks’ administration of omeprazole 40 mg × 1. They found differences in β-diversity and an increase in Streptococcaceae at the family level in both cirrhotic patients and controls and a reduction in Lachnospiraceae and Ruminococcaceae in cirrhotic patients. Serum gastrin was used as a proxy for the acid inhibiting effect of PPIs, and there was a positive correlation between serum gastrin and Streptococcaceae but not with other species.

Freedberg et al. [11] studied 12 healthy volunteers who all received omeprazole 40 mg × 2 for 4 weeks, and they were then randomized to either discontinue or continue PPIs for another four weeks. The study did not identify differences in α- nor β-diversity, but at the family level, there were increased abundances of Enterococcaceae, Streptococcaceae, Staphylococcaceae and Micrococcaceae, as well as a decrease in Clostridiaceae. The data were later reanalyzed by Jackson et al. [15], an analysis that was limited to the samples collected at 0 and 4 weeks. The methodology included filtering and analyses in QIIME and open reference clustering with a newer version of Greengenes v13_8. Jackson et al. found at the family level increased abundances of Streptococcaceae, Carnobacteriaceae, Burkholderiaceae and Corynebacteriaceae and at the genus level increases in *Streptococcus* and *Granulicatella* [15]. The different pipelines used for analysis (USEARCH and FastTree2) may also have generated different results. Consistent in both analyses was the increase in the family Streptococcaceae, which was predominant in the upper GI tract and was increased by more than 10-fold after PPI use [11]. The observed increases in families associated with *C. difficile* infection (CDI) (Enterococcaceae and Streptococcaceae) were consistent with the hypothesis that this could be the mechanism leading to CDI. 

Castellani et al. [16] investigated the fecal microbiota in infants who received esomeprazole 1 mg/kg/day for 4 weeks against GERD. They found that neither α- nor β-diversity changed significantly after treatment started, but α- and β-diversity were significantly increased after PPI cessation. This result may be a consequence of a PPI-induced prevention of the otherwise naturally occurring increase in microbial diversity in infants. At genus level, the relative abundances of *Lactobacillus* and *Stenotrophomonas* decreased while there was an increase in *Haemophilus*.

Otsuka et al. [17] included healthy volunteers starting vonoprazan, a K^+^ competitive acid blocker, as well as a group of healthy volunteers using lansoprazole 30 mg × 1 for four weeks, and the effects on the fecal microbiota were reported in a letter. Vonoprazan exerts an even more profound acid inhibiting effect than PPIs [18,19] and is marketed in some Asian countries. Vonoprazan 20 mg × 1 induced more complex alterations in the fecal microbiota than lansoprazole 30 mg × 1 for four weeks. The β-diversity in samples before and after acid inhibition differed for both vonoprazan and lansoprazole. Vonoprazan induced significant increases in the genera *Actinomyces*, *Rothia*, *Bacteroides*, *Granulicatella* and *Streptococcus* and significant decreases in the genera *Blautia* and *Coprococcus*. In contrast, lansoprazole induced significantly increased abundances of only two dominant genera, *Bacteroides* and *Streptococcus*.

Bajaj et al. [20] examined the fecal microbiota in patients with decompensated cirrhosis before and after treatment with omeprazole 40 mg × 1 for two weeks. They found a difference in β-diversity and an increase in oral-origin microbial taxa compared to baseline and a higher abundance of Streptococcaceae and Porphyromonadaceae families after PPI initiation. They also studied the fecal bacterial composition in 15 patients before and after withdrawal of omeprazole 20–40 mg × 1 and found an increase in the families Streptococcaceae, Veillonellaceae and Porphyromonadaceae.

Mishiro et al. [12] reported the changes in the fecal microbiota in ten healthy volunteers given esomeprazole 20 mg × 1 for four weeks; they found no differences in α-diversity nor β-diversity in fecal samples after PPI. The only reported change at the taxonomic level was an increased abundance of *Streptococcus*; however, such a change was not found in samples of saliva and from the periodontal pocket.

Reveles et al. [8] reported, after 14 days of omeprazole 20 mg, a difference in β-diversity and a reduction in the phylum Acinetobacteria as well as a reduction in the families Lachnospiraceae, Erysipelotrichaceae and Bifidobacteriaceae and an increase in Streptococcaceae, the latter being dominant in the oral microbiota [21]. 

Koo et al. [22] reported changes in the fecal microbiota in 34 healthy subjects, with an examination of fecal samples after omeprazole 20 mg × 1 for 7 days and then 7 days after cessation. They found a possible increase in α-diversity (assessed by richness but not by Shannon or Simpson) and no increase in β-diversity. At various taxonomic levels, there were increased abundances of the Bacilli class, in the Lactobacillales order and the family Streptococcaceae; at genus level, there were increased abundances of *Streptococcus* and *Veilonella* and of the species *Streptococcus vestibularis* and *Veilonella dispar*. The findings at several taxonomic levels seemed consistent with an increase in bacteria present in the normal flora of the oral cavity and GI tract.

Singh et al. [23] examined 15 healthy volunteers who were treated with omeprazole 20 mg × 1 for two weeks. There were no differences in α- nor β-diversity between the two time points. However, at the family level, there were increased abundances of Leuconostacaceae, Streptococcaceae and Pasteurellaceae, at genus level of *Cronobacter*, *Streptococcus*, *Klebsiella* and *Raoultella* and at species level of *Streptococcus pseudopneumoniae*, *Streptococcus salvarius* and *Streptococcus equinus*. The *Cronobacter*, *Klebciella* and *Raoultella* genera all belong to the enterobacteriacea family and are typically found in fecal samples, and the increase in Streptococcus at the family, genus and species levels suggests a change towards orally dominant flora.

Corazziari et al. [24] have published the largest study so far with 121 patients with GERD symptoms and a negative upper endoscopy before treatment with omeprazole 20 mg × 1 for four weeks. There were no differences in α- nor β-diversity between the two time points. At the species level, there were increased abundances of *Streptococcus salivarius*, *Streptococcus sinensis*, *Haemophilus parainfluenzae*, *Streptococcus dentisani*, *Streptococcus parasanguinis* and *Veillonella dispar*. These bacteria are all constituents of the normal oral bacterial flora.

### 3.3. Methodology and Heterogeneity

The field of microbiota research has evolved during the period from the first to the most recent study. Differences in DNA isolation protocols, sequencing methods, annotation and data analyses may all affect the results and cause diverging results. DNA extraction kits may isolate different bacteria to a dissimilar degree, and the choice of extraction kit has been reported to affect DNA yield but not diversity in saliva samples [25]. DNA extraction from fecal samples without mechanical and chemical lysis has resulted in the extraction of lower amounts of DNA from the Firmicutes, Bacteroidetes and Proteobacteria phyla [26]. It is also well recognized that the handling and preservation of fecal samples may affect the composition which is analyzed at a later stage [27], and these should ideally be standardized. In the reviewed studies, MoBio Powersoil or MoBio PowerFecal were used in three of the earlier studies and Qiagen DNA Stool or Qiagen DNease Powersoil were used in the three most recent studies, while for six studies, the DNA extraction was performed with a physical and chemical lysis step including bead beating but without a specific kit.

The different sequencing methods used may also have led to variation between the studies. Most studies used 16S rRNA sequencing performed on a MiSeq Illumina platform, while Roche 454-pyrosequencing was performed in three studies, and shotgun metagenomic sequencing was performed in one study.

The 16S rRNA gene consists of nine hypervariable regions, and within these regions, the DNA sequences vary, enabling the characterization of different taxa. There were considerable differences in which 16S regions were targeted, as the V3–V4 regions were chosen in three recent studies and the V1–V2, V3–V5, V4 and V6–V7 regions were all chosen in one study each, while in one study, the region was not specified. The choice of 16S primers significantly affects the sequencing results, as for instance the V5 region enables the identification of bacteria at the phylum level whereas the Shannon diversity index is higher when targeting the V3/V4 region [28]. The sequencing of V3–V4 may be combined to delineate bacteria [29] and accommodates common sequencing technologies that have been more frequently used in the latter part of the publishing period; however, reliable sequencing at the species level has still been questioned [30,31].

Several different pipelines for analyses of sequencing data were used in the thirteen studies, including QIIME, QIIME2, USEARCH and Dada2. The filtering of sequencing data is performed in all pipelines, and this removes rare taxa with a low contribution to the overall signal, which will reduce the dimensionality of the data with limited loss of information [32].

The limitations of the field also include that many of the studies had a small sample size. Although sound meta-analyses of the existing studies are not feasible, general trends such as changes in diversity may be assessed. There are currently no studies of the effect of PPIs on the small intestinal microbiome, and such studies should ideally be conducted with surgical sampling [33] to avoid contamination from gastric juice or colonic flora inevitably caused by endoscopic sampling. There are also major differences between the fecal and mucosal microbiota in the colon [34], and although the sample collection is more challenging, the mucosa-adjacent changes in microbiota may be more important in the pathogenesis of many diseases. Changes in diversity and composition per se may be an epiphenomenon that accompanies important changes in the interaction between the microbiota and the host. Sequencing-based studies of samples from the stomach or small intestine examine composition and not the absolute number of bacteria. It is possible that the development of some diseases which are caused by microorganisms depends on the absolute number of pathogenic bacteria and their products more than their relative proportion, which may not reflect major changes in absolute numbers. Over 50 years ago, it was found that patients with gastric hypoacidity had a higher number of colony forming units (CFUs)/mL gastric juice, reaching a magnitude of a 1000-fold difference [35]. Although non-culture-based studies have obvious advantages and outnumber old studies, some information may be missed by only focusing on relative composition.

**Table 1:** Changes in the gastrointestinal microbiota induced by proton pump inhibitors in experimental trials.

Study Design	Participants (n) PPI (Generic Drug, Dose and Duration) Microbiota (DNA Extraction Kit, DNA Region Targeted, Sequencing Platform, Quality Filtering, Reference Database)	Diversity	Microbiota Alterations on Taxonomic Level							
				Phylum	Class	Order	Family	Genus	Species	Ref.
Prospective open label Healthy volunteers	n = 9 Omeprazole 20 mg × 1 or ×2/day, 4 weeks 16S rRNA sequencing of faecal samples MoBio Powersoil V3–V5 MiSeq Illumina platform USEARCH/UPARSE Greengenes	Reduced α-diversity (OTU count)		-	-	-	-	-	-	[13] Seto 2014
Prospective open label Patients with GERD symptoms (normal upper endoscopy n = 2, esophagitis n = 4, Barrett’s n = 2)	n = 8 Lansoprazole 30 mg × 2/day, 8 weeks 16S rRNA pyrosequencing of oesophagal biopsies and gastric fluid MoBio Powersoil V6–V7 Roche 454-pyrosequencing QIIME/UCLUST RDP	Difference in β-diversity UniFrac (uw)	↑ in biopsies ↓ in biopsies ↑ in gastric fluid ↓ in gastric fluid	- - - -	- - - -	- - - -	Clostridaceae, Lachnospiraceae Microccocaceae Actinomycetaceae Comamonadaceae Erysipelotrichaceae Moraxellaceae Flavobacteriaceae Comamonadaceae Methylobacteriaceae	- - - -	- - - -	[10] Amir 2014
Prospective open-label trial. Compensated cirrhosis and controls	n = 30 Omeprazole 40 mg/day, 2 weeks 16S rRNA pyrosequencing of faecal samples Extraction NS Universal primers for bacteria Roche 454-pyrosequencing QIIME, RDP	Difference in β-diversity UniFrac (w) and Bray-Curtis	↑	-	-	-	Streptococcaceae (cirrhosis 0.0 → 8.9, controls 0.2 → 5.7) ^a^	-	-	[14] Bajaj 2014
Prospective, block randomized Healthy volunteers	n = 12 Omeprazole 40 mg × 2/day, 4 or 8 weeks 16SrRNA sequencing of faecal samples Mo Bio PowerFecal V4 Illumina MiSeq Mothur/USEARCH, Greengenes QIIME, Greengenes	No difference in α-(Shannon) or β-diversity (UniFrac, w and uw)	↑ ↓ ↑	- -	- -	- -	Enterococcaceae, Streptococcaceae, Staphylococcaceae, Micrococcaceae Clostridiaceae Streptococcaceae, Carnobacteriaceae, Burkholderiaceae, Corynebacteriaceae	- - *Streptococcus*, *Granulicatella*	- -	[11] Freedberg2015 [15] ^e^ Jackson 2016
Prospective open-label trial. Infants < 1 y with GERD	n = 12 Esomeprazole 1 mg/kg/day, 4 weeks 16S rRNA sequencing of faecal samples ^f^ Extraction NS V1-2 Illumina MiSeq QIIME/UCLUST Greengenes	Reduced α-diversity during treatment (observed OTU, Chao1) Difference in β-diversity UniFrac (uw)	↑	-	-	-		*Haemophilus * (0 → 2.3 × 10^−5^) ^b^ *Lactobacillus* (2.6 × 10^−4^ → 2.9 × 10^−5^) ^b^, *Stenotrophomonas* (5.1 × 10^−5^ → 1.6 × 10^−6^) ^b^		[16] Castellani 2017
Prospective open-label trial. Healthy volunteers	n = 11 Lansoprazole 30 mg/day, 4 weeks 16SrRNA sequencing of faecal samples Illumina Miseq Other parameters NS		↑	-	-	-	-	*Bacteroides* (7.85 → 18.5) ^c^, *Carnobacterium* (0.005 → 0.014) ^c^, *Streptococcus* (1.35 → 8.5) ^c^, *Oribacterium* (0.001 → 0.002) ^c^		[17] Otsuka 2017
Prospective open-label trial. Decompensated cirrhosis PPI withdrawal	n = 15 Omeprazole 40 mg/day, 2 weeks 16S rRNA pyrosequencing of faecal samples Roche 454-pyrosequencing Other parameters NS	Difference in β-diversity UniFrac (w) and Bray-Curtis	↑ (after initiation) ↑ (before withdrawal)	-	-	-	Streptococcaceae Porphyromonadaceae Streptococcaceae Veillonellaceae Porphyromonadaceae ^e^			[20] Bajaj 2018
Prospective open label Healthy elderly (age ≥ 60 years)	n = 24 Omeprazole 20 mg/day, 2 weeks 16S rRNA sequencing of faecal samples Extraction NS V4 Illumina MiSeq, Morthur, Greengenes	No difference in α-diversity (richness, Shannon) Difference in β-diversity UniFrac (uw)	↑ ↓	- Actinobacteria (4.25 → 2.35) ^a^	- -	- -	Streptococcaceae (1.49 → 5.93) ^b^ Lachnospiraceae (33.40 → 28.60) ^b^ Erysipelotrichaceae (4.09–2.74) ^b^ Bifidobacteriaceae (2.83 → 1.39) ^b^	- -	- -	[8] Reveles 2018
Prospective open label Healthy volunteers	n = 10 Esomeprazole 20 mg/day, 4 weeks 16S rRNA sequencing of salvia, periodontal fluid and faecal samples Extraction NS V3–V4 Illumina MiSeq QIIME/USEARCH/UCLUST Greengenes	Reduced α-diversity (Shannon) in salvia after PPI	↑ in saliva ↑ in periodontal pocket ↑ in faeces	- - -	- - -	- - -	- - -	*Veillonella*, *Neisseria*, *Leptotrichia* *Streptococcus*	- - -	[12] Mishiro 2018
Prospective open label Healthy volunteers	n = 34 Omeprazole 20 mg/day, 7 days 16SrRNA sequencing of faecal samples QIAamp DNA Stool Mini kit V3–V4 Illumina MiSeq, QIIME, RDP	Increased richness, no difference in other α-diversity indices (Shannon, Simpson), no difference in β-diversity (Bray-Curtis, Sørensen Dice)	↑	-	Bacilli	Lactobacillales	Streptococcaceae	*Streptococcus*, *Veillonella*	*Streptococcus vestibularis*, *Veilonella dispar*	[22] Koo 2019
Prospective double blind, placebo-controlled Healthy volunteers	n = 15 Omeprazole 20 mg/day, 2 weeks Shotgun metagenomic sequencing of faecal samples Qiagen DNeasy Powersoil Illumina HiSeq, Sunbeam pipeline/Trimmomatic, Kraken	No difference in α-diversity (Shannon, Simpson, Chao1, Fisher) No difference in β-diversity	↑	-	-	-	Leuconostacaceae ^d^, Streptococcaceae, Pasteurellaceae	*Weissella*, *Cronobacter*, *Streptococcus*, *Klebsiella*, *Raoultella*	*Streptococcus pseudopneumoniae*, *Streptococcus salvarius*, *Streptococcus equinus*	[23] Singh 2022
Prospective double blind, placebo-controlled GERD symptoms and negative upper endoscopy	n = 121 Omeprazole 20 mg/day, 4 weeks 16S rRNA sequencing of faecal samples Qiagen DNA Stool Mini V3–V4 USEARCH, QIIME2, Dada2, Greengenes	No difference in α-(Shannon, observed OTUs, Faith) or β-diversity (Bray Curtis, UniFrac (w))	↑	-	-	-	-	-	*Streptococcus salivarius*, *Streptococcus sinensis*, *Haemophilus parainfluenzae*, *Streptococcus dentisani*, *Streptococcus parasanguinis*, *Veillonella dispar*	[24] Corazziari 2023

Only results of statistical significance of *p* < 0.05 are included; unclassified taxa are not included. ^a,b,c^ Changes in median, mean and unspecified relative abundance, respectively. ^d^ Leuconostacaceae family has been reclassified into *Lactobacillaceae* [36]; ^e^ reanalyses of data from [11] ^f^ examined after PPI withdrawal; ↑: increased; ↓: decreased; -: not reported; uw: unweighted; w: weighted; NS: not specified; DNA extraction based on physical and chemical lysis steps including bead beating.

## 4. Discussion

In these thirteen identified experimental trials, PPI use was associated with moderate changes in the microbiota of the upper and lower GI tract. The effects were seen on alterations in diversity and richness in some of the studies, while a larger proportion of the studies detected alterations at various taxonomic levels. The general finding in this review is that PPI use caused an increase in bacteria normally found in the oral microbiota in both the upper and lower GI tract. There seemed to be a consistent increase in the oral flora along the axis Streptococcaceae, Streptococcus at genus level and various *Streptococcus* at species levels, as well as Veillonellaceae, *Veillonella* and *Haemophilus*. Several other changes were found in single studies, and this inconsistency may be related to the methodological differences and heterogeneity described in Section 3.3, as well as differences in patient populations and the low number of participants in many of the studies.

A previous systematic review reported changes in the microbiome of the digestive tract in publications up to 2019 in both observational and experimental studies [37]. In the current review, we have focused on the changes found in interventional studies only since this eliminates several confounders. In addition, two additional studies that included altogether 136 patients [23,24] have been published since 2019. The current report is more focused on observations of higher methodological quality and therefore adds new information compared to previous reviews.

Alterations in the GI microbiome have been associated with various diseases including inflammatory bowel disease [38,39], neurological disease [40], diabetes [41], liver disease [42] and cardiovascular disease [43]. The potential side effects of PPIs also include increased risks of some malignancies, nutrient deficiencies [44] and infections [45], and they may also affect the course of inflammatory diseases [46]. There are also reports that PPI use is associated with increased fecal calprotectin in multivariable analyses [47] and PPI use is associated with an increased risk of immune check point inhibitor-induced colitis [48]. Several side effects of PPIs may be mediated by microbiome alterations, and at the population level, PPI use is the single factor that influences fecal microbial composition to the largest extent [6,7]. PPIs are misused in many instances. PPI trials against unspecific symptoms without prior investigations may have a high placebo effect, leading to long-term use without any benefit, and cannot be justified [49]. It is therefore of considerable interest to understand how PPIs affect the microbiome in detail.

It is well known that the bactericidal effect of gastric juice is markedly diminished when the intragastric pH increases to above 4 [50]. Several factors may influence the effects PPIs may have on the microbiome. PPIs administered once daily often increase the intragastric pH to above 4 for 12 h per day; however, there is considerable variation between the different PPIs [19], i.e., the potency of PPIs differs between the various PPIs [19,51], and different doses of PPIs were used in the thirteen studies in this review. In studies that found differences in β-diversity, participants used PPIs with a more pronounced effect on intragastric pH (lanzoprazole 30 mg × 2, omeprazole 40 mg × 1), and the different degree of acid inhibition as well as the duration of use may explain some of the discrepancies between the study findings. The patients’ age may also influence the degree of PPI-induced changes in the microbiota, as older patients may have reduced acid secretory capacity due to the higher prevalence of some degree of oxyntic atrophy [52]; the microbiota in elderly patients has its own characteristics and may be more vulnerable [53]. Reveles [8] found that PPIs induced differences at the phylum and family level in elderly patients (mean age 71 years), while Corazziari [24] did not find differences (mean age 48); both studies used omeprazole 20 mg × 1, although the sequencing and analysis pipelines differed. In addition to the impact PPIs have on the microbiome, the composition of food affects the fecal bacterial composition after a short time [54]. PPI use with improvement in upper GI symptoms may therefore have led to dietary changes, causing alterations in the fecal microflora not caused by PPI treatment per se but by the change in diet. However, the alterations in the microbiota of the upper GI tract should not be affected by diet composition to a similar extent.

Three of the studies enrolled participants that may not be representative of the majority of PPI users. Patients with liver cirrhosis [14,20] may be more vulnerable to PPI-induced changes in the GI microbiota, either related to an attenuated immune response or due to portal hypertension accompanied by increased intestinal permeability seen in advanced cirrhosis [55]. However, the finding of PPI-induced increased abundances of Streptococcaceae and Veillonellaceae corresponds to changes at several taxonomic levels in studies of other study populations. One study included infants aged <1 year [16], and since the GI microbiota in infants undergoes a considerable development in the first year of living [56,57], the findings in infants may not be extrapolated to adult populations. It is still of interest that the authors reported lower α- and β-diversities as well as higher abundances of oral bacteria at the species level during PPI treatment, which is in line with general tendencies in adult study populations.

The increase in components of oral bacterial flora, especially *Streptococcus* spp., may represent an unfavorable gut microbial alteration or dysbiosis, which may increase the risk of many diseases with a high prevalence globally. A large cross-sectional study found increased fecal abundance of *Streptococcus* spp. to be associated with coronary arteriosclerosis and systemic markers of inflammation, independent of cardiovascular risk factors [58]. Systematic reviews and meta-analyses have also found an increased abundance of streptococcus in patients with hypertension [59] and NAFLD [60]. Additionally, streptococcus enrichment is found in the gastric biopsies and feces of patients with gastric cancer [61,62]. 

In the current article, we have focused on interventional studies in order to reduce confounders that limit the internal validity of observational studies. However, experimental studies often have a low number of subjects, limiting the external validity of findings from sequencing studies. Of the thirteen identified studies, only four included more than 20 subjects, and future studies of larger populations as well as the use of validation cohorts are necessary. There is a need for larger studies that investigate changes in pronounced acid inhibition in various patient cohorts, particularly in groups that may be frail or with a microbiota that could be more vulnerable to the effects of PPI use. So far, there are no studies that have investigated the mucosa-adjacent microbiota found in colorectal biopsies, which may potentially be far more relevant for disease risk than the luminal flora. The relative paucity of studies of the microbiota of the upper GI tract is noteworthy and may be related to the relative ease of collecting fecal samples compared to gastric juice or gastric mucosal biopsies. However, despite logistic and methodological challenges, there is a need for experimental studies with a larger number of patients investigating PPI-induced changes within the upper GI tract. There is also a need for studies that do not only consider the relative composition of the microbiota but that also quantify the absolute increase in bacteria following the inhibition of gastric acid secretion. Finally, some diseases may be caused not by the presence of bacteria per se but by the metabolites produced by bacteria that may be present at higher concentrations following PPI use, and future studies should, to a larger extent, correlate microbiota to the metabolome. Two ongoing studies will provide more evidence about the PPI-induced alteration in microbiota and the association with clinically relevant endpoints in patients with cirrhosis [63] and in GERD patients [64].

## 5. Conclusions

PPIs significantly impact the GI microbiome from the esophagus to feces. Current evidence from experimental studies demonstrates that the reduction in gastric acidity reduces the defense against swallowed bacteria and that pathogenic species may more easily have a negative impact on the organism. However, the long-term effects of PPI use on health and disease mediated by microbial changes are largely unknown.

## Data Availability

No original data were presented.
